# Research Literacy in Residency: Development, Implementation, and Outcomes for the Psychiatry Research Methods and Scholarship (PReMS) Curriculum

**DOI:** 10.1007/s40596-026-02309-9

**Published:** 2026-03-18

**Authors:** Susan K. Mikulich-Gilbertson, Joseph T. Sakai, Emmaly Perks, Crystal Natvig, Devika Bhatia, Kimberly Slavsky, C. Neill Epperson

**Affiliations:** University of Colorado Anschutz Medical Campus, Aurora, CO USA

**Keywords:** Research literacy, Residency curriculum, ACGME competencies

## Abstract

**Objective:**

Development, implementation, and outcomes are described for the Psychiatry Research Methods and Scholarship (PReMS) curriculum targeting research literacy and scholarship during second year post graduate (PGY-2) residency training.

**Methods:**

PReMS development at the University of Colorado Department of Psychiatry (UC-DOP) incorporated expertise from residency leadership, pedagogy, and biostatistics and was fine-tuned during 5 years of implementation. Content addressed Accreditation Council for Graduate Medical Education core competencies in research literacy and scholarship. Faculty-authored publications from UC-DOP highlighted research aspects including different study designs, methods, populations, sources of bias, and statistical analyses. Residents chose a paper and led its discussion; lecture materials supported the concepts addressed. Faculty authors were invited to sessions when their papers were presented to meet residents, answer questions, and stimulate discussion. The course director developed an assessment for administration before and after PReMS.

**Results:**

Across five resident cohorts (*n* = 66), an increasing number and proportion of invited faculty authors attended sessions discussing their papers (93% overall). After PReMS, residents’ research literacy scores improved significantly (*p* < 0.0001) by 17% on average after accounting for cohort; ratings of the importance of research to psychiatrists were unchanged.

**Conclusions:**

Preliminary results suggest PReMS is an effective curriculum to improve research literacy in residency and thereby advance proficiency in critical evaluation of the medical literature, a crucial skill for all physicians to become life-long learners. PReMS innovatively uses resident-led discussions of faculty-authored publications to make pedantic methodological material more interesting and to provide exposure to the implementing program’s researchers, potentially enhancing scholarship mentoring opportunities.

Beginning in 2018, the University of Colorado Department of Psychiatry (UC-DOP) residency program in a public medical school invested substantial time and resources into developing and implementing a curriculum targeting the relevant Accreditation Council for Graduate Medical Education (ACGME) Core Competencies [1, 2] in research literacy and scholarship. This report describes that process and 5-year outcomes of the Psychiatry Research Methods and Scholarship (PReMS) curriculum.

ACGME states “All residents must be educated in research literacy and in the concepts and process of evidence-based clinical practice to develop skills in question formulation, information searching, critical appraisal, and medical decision-making” [2, 4.15.c]. For decades, others have been noting and proposing ways to address gaps in residency education on research literacy (e.g., [3–9]). As a foundation to research methodology, insufficient biostatistics knowledge among clinicians, in particular, has been widely recognized (e.g., [10–12]).

ACGME further asserts: “Residents must participate in scholarship” [2, 4.15], “The program must provide residents with opportunities for research and development of research skills for residents interested in conducting research in psychiatry or related fields” [4.15.a], and “The program must provide interested residents access to and the opportunity to participate actively in ongoing research under a mentor” [4.15.b]. In their editorial “Research Training and Education at the Crossroads”, Balon and colleagues acknowledge that these may be difficult to achieve for some residency programs [5].

The UC-DOP program addresses ACGME scholarship competencies by requiring each resident to complete a mentored scholarly project. The PReMS curriculum was designed to support that requirement while advancing research literacy. PReMS objectives are to familiarize learners with the defining characteristics of observational and experimental study designs, including their advantages and vulnerabilities; provide exposure to common research methodology including statistical procedures, improve their critical thinking, and advance their proficiency in the evaluation of research publications; and provide connections to faculty mentors and tools for the successful completion of a scholarly project. Additionally, concerted effort was made to make the often-perceived pedantic methodological material more interesting and relevant. It was hypothesized that after PReMS, residents’ research literacy knowledge scores would improve, and their ratings of the importance of research to psychiatrists and likelihood of being involved in research would increase.

## Methods

**Fig. 1:**
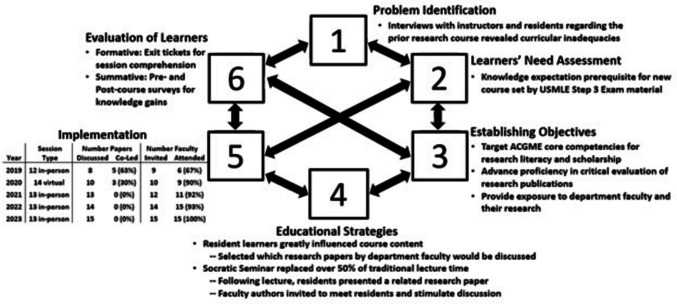
Application of Kern’s six-step approach to curriculum development for medical education to the Psychiatry Research Methods and Scholarship (PReMS) curriculum. Abbreviations used: USMLE, United States Medical Licensing Examination; ACGME, Accreditation Council for Graduate Medical Education

First, the PReMS curriculum development and changes that occurred are described and summarized in Fig. 1. PReMS incorporated expertise from psychiatry residency leadership and pedagogy, along with research methods and biostatistics, using an established six-step process of curriculum development [13] and Backwards Design model [14].

Prior to 2019, the UC-DOP program addressed research literacy with a single session provided in the third year of postgraduate training (PGY-3). Interviews with instructors of that course (PhD psychologists) and with prior resident learners revealed an instructor/learner mismatch concerning the course objectives and useful content (Fig. 1, Step 1). As a biostatistician and non-clinician, the PReMS course director (first author SMG) learned from program leadership that the key reason residents needed research literacy was to understand published scientific findings and to determine the applicability of those findings to their clinical practices. This became the central tenet for PReMS.

The UC-DOP program wanted to shift research education to PGY-2. Based on their requirement that residents complete the United States Medical Licensing Examination (USMLE) Step 3 by December of PGY-2, the course director leveled PReMS material to be appropriate for learners who had recently completed or were in preparation for Step 3. Incoming PGY-2 residents were informed that familiarity with exam topics such as prevalence, variable types, hypotheses, and *p*-values [15] was assumed (Fig. 1, Step 2).

PReMS was designed to advance proficiency in critical evaluation of research and provide exposure to UC-DOP research faculty (Fig. 1, Step 3), but minimize lecture time and increase learner interest by using resident-led Socratic Seminar discussions of resident-chosen research publications to exemplify key methodology (Fig. 1, Step 4). The course director used UC-DOP faculty-authored publications to highlight different aspects of research such as study designs (e.g., cohort, quasi-experimental), methods (e.g., medication trial, survey), populations (e.g., adolescents, mice), and statistical analyses. In 2021, the course director began creating a survey of potential publications for discussion within each study design category for incoming PGY-2 residents to indicate papers that they wanted to hear discussed and faculty authors they wanted to meet, enabling learners to influence course content. Once the papers were chosen, they were slotted into the class schedule so that lecture materials complemented and supported concepts addressed in that session’s paper. Residents each signed up to provide an overview and lead discussion of a paper. Faculty authors were invited to attend sessions during which their papers were presented to meet the residents and stimulate further discussion (Fig. 1, Step 4). This supported the UC-DOP program’s requirement that all residents identify a mentor and develop a scholarly project proposal by December of PGY2.

With each implementation, PReMS has been refined, and the current curriculum is under review at the American Association of Directors of Psychiatric Residency Training (AADPRT). In 2020, with the COVID-19 pandemic, PReMS was held virtually, which led residents to create slides for their paper discussions. That practice worked well and continued after the return to in-person sessions. In 2021, the course director began requiring the slides be emailed by the day prior to presentation for her review and offering to meet with the presenter to answer questions. This has allowed her to recommend adjustments to presentation length and content and increase residents’ confidence during their presentations.

Figure 1, Step 5 tabulates key changes in implementations by year. Initially, some residents co-led paper discussions with a peer. Since summer 2021, each resident has individually led a paper, maximizing exposure to more topics and faculty such that PReMS in 2024 consisted of 13 fifty-minute sessions with 15 resident-led paper discussions. Lecture material has been consolidated to allow time for some sessions to include the discussion of 2 papers (each approximately 20 min).

Across cohorts, faculty authors have attended 93% of the sessions to which they were invited; for some sessions, multiple authors attended. To date, 33 faculty authors have attended one or more PReMS sessions, and the number and proportion of invited faculty participating has increased from 6 faculty attending of 9 invited in 2019 to 15 faculty attending of 15 invited in 2023 (Fig. 1, Step 5).

To assess the impact of PReMS, 2 forms of evaluation were utilized (Fig. 1, Step 6) per pedagogy recommendations. Lectures concluded with an Exit ticket querying comprehension. Answers to these questions were discussed at the beginning of the next session.

Lacking an appropriate validated assessment of research literacy, the course director developed a survey with 10 multiple choice questions administered pre- and post-PReMS. Items queried concepts covered in class deemed important to evaluating a study’s results credibility (e.g., biases, power), or that reference methodology noted to be misused in the literature (e.g., logistic regression to estimate risk for a condition that is not rare, mediation). Change in the cumulative score across items and change in percent correct on each item after PReMS assessed knowledge acquisition. Using scales ranging from 1 (lowest) to 10 (highest), learners were also asked to rate the importance of research to psychiatry and to their careers as psychiatrists, as well as the likelihood of their involvement in research in their careers. Initially, the assessment was distributed in paper form (available upon request) during the first and final sessions. In 2020, a version was developed in REDCap (Research Electronic Data Capture, a secure, HIPAA-compliant web-based application) and emailed to the residents via a link. Participation was voluntary and encouraged. This protocol was exempted from Institutional Review Board (IRB) oversight by the university’s IRB.

Mixed model analyses of variance (ANOVAs) that are advantageous in their ability to include subjects with missing data [16] were used to evaluate the number of correct responses on the survey before and after receiving PReMS, and changes in the rating scales. Because of program and curriculum changes throughout implementation, potential cohort effects were evaluated. Fixed effects of cohort (1 to 5), time (pre, post), and the cohort by time interaction, as well as a random subject effect, were specified. If the cohort by time interaction was nonsignificant, it was removed and the model re-run.

Generalized linear mixed models with logit links were used to evaluate correct responses for each of the 10 research literacy items and odds ratios for correct response post-PReMS compared to pre-PReMS after adjusting for cohort were estimated.

## Results

Since July of 2019, 5 cohorts have completed PReMS. For Cohorts 1 to 3, 13/13 residents completed the pre-assessment each year, and in terms of post-assessment, 8/13 in Cohort 1 and 13/13 in Cohort 2 and Cohort 3 completed. For Cohort 4, 9/13 completed the pre-assessment and 13/13 completed the post-assessment. For Cohort 5, the residency program grew and 11/15 completed the pre-assessment and 10/15 completed the post-assessment.

Table 1 shows results for each research literacy outcome from the survey. In statistical models for all outcomes, the cohort by time interaction was nonsignificant and removed. As hypothesized, cumulative knowledge scores (number of correct items of 10 total) improved modestly but significantly (*p* < 0.0001) after PReMS (Mean = 5.2) compared to before (Mean = 3.5), after adjusting for cohort (Table 1, Part A).

**Table 1:** Research literacy results before and after the Psychiatry Research Methods and Scholarship (PReMS) curriculum in 5 cohorts of residents (*N* = 66 total) from mixed model analyses of variance (A, C) and generalized linear mixed models (B)

	**Pre-PReMS** **Mean (SE)**^**a**^ ***N* = 59**	**Post-PReMS** **Mean (SE)** ***N* = 57**	**Cohort** **Effect**	**Time** **Effect**	
**A. Score: Number of Items Answered Correctly of 10 Total**	3.48 (0.19)	5.19 (0.20)	NS^b^	*p* < 0.0001	
**B. Items Querying Statistical and Research Methods**	**Pre-PReMS** ***N* correct (%)**	**Post-PReMS *N* correct (%)**	**Cohort** **Effect**	**Time** **Effect**	**aOR (95% CI)**^**c**^** Post versus Pre PReMS**
1) De-identifying a test is to prevent which type(s) of bias	20 (33.9%)	31 (54.4%)	NS	*F*_1,49_ = 4.83 *p* = 0.033	2.43 (1.08, 5.46)
2) With ANOVA^d^, which test(s) for evaluating relationships between outcomes and predictors, covariates, confounders, moderators and independent variables are different	9 (15.3%)	26 (45.6%)	NS	*F*_1,49_ = 11.2 *p* = 0.002	4.75 (1.87, 12.1)
3) Causes of erroneous results for associations among outcomes	50 (84.8%)	49 (86.0%)	NS	NS	1.02 (0.29, 3.58)
4) Study design(s) in which the exposure and outcome variables are measured simultaneously	37 (62.7%)	48 (84.2%)	NS	*F*_1,49_ = 7.97 *p* = 0.007	4.32 (1.52, 12.3)
5) Identify types of paired study design(s) of an educational intervention	8 (13.6%)	10 (17.5%)	NS	NS	1.59 (0.53, 4.75)
6) Identifying types of biases that could impact a research study	40 (67.8%)	40 (70.2%)	NS	NS	1.17 (0.49, 2.78)
7) Identifying statistical procedure(s) for estimating risk of predictors of an outcome that is not rare (prevalence greater than 15%)	3 (5.1%)	13 (22.8%)	*F*_4,49_ = 2.74; *p* = 0.04	*F*_1,49_ = 9.11 *p* = 0.004	13.3 (2.37, 74.1)
8) Characteristics of intent to treat (ITT) design and ITT analysis	13 (22.0%)	24 (42.1%)	NS	*F*_1,49_ = 5.09 *p* = 0.029	2.65 (1.11, 6.31)
9) When describing results from a study that compares two groups, identify condition(s) when reporting the study’s power is important	6 (10.2%)	28 (49.1%)	NS	*F*_1,49_ = 17.1 *p* = 0.0001	8.83 (3.06, 25.4)
10) Conditions under which a variable may be a mediator of the relationship between an exposure variable and an outcome	21 (35.6%)	27 (47.4%)	NS	NS	1.59 (0.72, 3.50)
**C. Ratings Regarding Research in Psychiatry**	Mean (SE)	Mean (SE)	**Cohort** **Effect**	**Time** **Effect**	
1) On a scale of 1 (very unimportant) to 10 (extremely important), How important is research to the field of psychiatry in general?	9.09 (0.17) [Median = 10]^e^	8.90 (0.18) [Median = 10]	NS	NS	
2) On a scale of 1 (very unimportant) to 10 (extremely important), How important will research be to your career as a psychiatrist?	6.43 (0.30)	6.20 (0.30)	NS	NS	
3) On a scale of 1 (very unlikely) to 10 (extremely likely), how likely do you think it is that you will be involved in one or more aspects of conducting research in your career as a psychiatrist?	5.66 (0.36)	5.06 (0.37)	NS	NS	

^a^*SE*, standard error

^b^*NS*, nonsignificant, *p* > 0.05

^c^*aOR (95% CI)*, adjusted odds ratio (95% confidence interval) where adjustment is for cohort

^d^*ANOVA*, analysis of variance

^e^Median is also reported because outcome was not normally distributed; nonsignificant result was confirmed with Wilcoxon signed-rank test

Table 1 Part B lists each survey item with the correct answer and reports the number with correct responses pre- and post-PReMS. In hindsight, the test seemed overly difficult for this population. Results from the generalized linear mixed models showed that only one item had a significant cohort effect (item 7) and 6 items showed significant improvement following PReMS (items 1, 2, 4, 7, 8, 9), such that a resident’s odds of a correct response after adjusting for cohort in those models ranged from 2.4 to 13.3 times higher after receiving PReMS compared to before receiving PReMS.

Contrary to hypotheses, none of the 3 ratings on aspects of research in psychiatry changed significantly following PReMS (Table 1 Part C). Residents rated the importance of research to psychiatry consistently very highly such that the outcome was not normally distributed, so medians were included (item 10 at pre and post), and nonsignificant results from the mixed model ANOVA were confirmed with a Wilcoxon signed rank test.

## Discussion

The PReMS curriculum was carefully developed to target the aspects of research literacy that are essential for all residents, regardless of interest in a career involving research. PReMS emphasizes the knowledge and skills necessary to interpret the research literature and evaluate the applicability of published results to a psychiatrist’s specific clinical practice. As noted by Forehand and colleagues [6], mental health research is characterized by challenges such as designing appropriate control conditions for psychotherapy trials [17], making skills in the design (for psychiatrist researchers) and interpretation of published results (for *all* psychiatrists) even more crucial.

Across 5 cohorts, significant modest gains in research literacy were noted after PReMS. However, limitations of this preliminary study include small sample sizes, methodological adaptations during implementations, inability to evaluate potential confounds beyond adjusting for potential cohort effects in models, and most importantly, lack of an appropriate tool for assessing research literacy. The survey that was developed lacks validation. When administration occurred via email, residents were not specifically prohibited from discussing answers. The goal was for correct answers to the technical questions covering broad areas to indicate adequate research literacy for evaluating the quality of published studies, but the survey provides an indirect measurement of that skill at best. Furthermore, residents missed sessions for vacations, illness, parental leave, night float, etc. Lecture materials were available but there was no requirement to make up work and absenteeism may have impacted improvement in survey scores. Ideally, PReMS success would be directly measured by residents’ ability to critique published research—a potential evaluation area for future development. Other research literacy residency curriculums assessed gains in residents’ confidence and their course satisfaction (e.g., [18, 19]), but none to our knowledge address research literacy knowledge and skills acquisition, perhaps also due to lack of an appropriate assessment.

PReMS’s diminished emphasis on traditional lecture time was purposeful to avoid “Death by PowerPoint” [20], considering the commonly perceived pedantic nature of methodological material. Most sessions illustrate research methodology with presentations and discussions led by the resident learners of publications that are authored by faculty from the residency program. This format conveys the advantages of a journal club approach, but with the added benefit of having the paper author accessible. PReMS thereby innovatively provides knowledge of, and connection to, that program’s research and researchers, facilitating mentoring and scholarly project opportunities. Except for the course director, the burden of implementing PReMS for other program faculty is relatively minimal (about 20 min to attend a presentation that involves discussion of work with which they have authored and are already familiar). It is gratifying to see one’s research presented, and the faculty authors were consistently complementary and often requested a copy of the slides from the residents. Multiple faculty expressed that they wished they had received such a curriculum during residency.

In conclusion, preliminary results indicate the utility of our approach to advancing research literacy in residency. The PReMS curriculum is currently under review at AADPRT with complete lecture slides and research publication examples, as well as flexible implementation options and formatting simplifications included to accommodate potential obstacles such as time limitations and smaller residency programs with few research faculty. The hope is that these provisions might facilitate more widespread dissemination of a curriculum for research literacy or even that aspects of the approach used here might be beneficially applied to other areas of ACGME requirements.

## Data Availability

De-identified data are available upon request to the corresponding author.

## References

[1] ACGME Common Program Requirements (Residency). Effective July 1, 2025. https://www.acgme.org/globalassets/pfassets/programrequirements/2025-reformatted-requirements/cprresidency_2025_reformatted.pdf. Accessed May 10, 2025.

[2] ACGME Program Requirements for Graduate Medical Education. Effective July 1, 2025. https://www.acgme.org/globalassets/pfassets/programrequirements/2025-reformatted-requirements/400_psychiatry_2025_reformatted.pdf. Accessed May 10, 2025.

[3] Roane DM, Inan E, Haeri S, Galynker II. Ensuring research competency in psychiatric residency training. Acad Psychiatry. 2009;33:215–20. 10.1176/appi.ap.33.3.215. 19574518 10.1176/appi.ap.33.3.215

[4] Hamoda HM, Bauer MS, DeMaso DR, Sanders KM, Mezzacappa E. A competency-based model for research training during psychiatry residency. Harv Rev Psychiatry. 2011;19(2):78–85. 10.3109/10673229.2011.565249. 21425936 10.3109/10673229.2011.565249

[5] Windish DM. Brief curriculum to teach residents study design and biostatistics. Evid Based Med. 2011;16:100–4. 10.1136/ebm.2011.04.0011. 21771818 10.1136/ebm.2011.04.0011

[6] Forehand JA, Levis M, Watts BV, Finn CT, Shiner B. Research literacy for psychiatry residents: a 10-session curriculum using a problem-based learning approach. Acad Psychiatry. 2022;46:504–9. 10.1007/s40596-021-01487-y. 34046863 10.1007/s40596-021-01487-y

[7] Cozza EM, Shankman SA. Integrating NIMH’s research domain criteria (RDoC) initiative into psychiatry research training. Acad Psychiatry. 2022;46:522–7. 10.1007/s40596-021-01547-3. 34642858 10.1007/s40596-021-01547-3 PMC12206478

[8] Balon R, Morreale MK, Louie AK, Aggarwal R, Guerrero APS, Coverdale J, et al. Research training and education at the crossroads. Acad Psychiatry. 2022;46:417–20. 10.1007/s40596-022-01682-5. 35854176 10.1007/s40596-022-01682-5 PMC9296110

[9] Stevenson MD, Smigielski EM, Naifeh MM, Abramson EL, Todd C, Li STT. Increasing scholarly activity productivity during residency: a systematic review. Acad Med. 2017;92(2):250–66. 10.1097/ACM.0000000000001169. 27049539 10.1097/ACM.0000000000001169

[10] Swift AL, Miles S, Price GM, Shepstone L, Leinster S. Do doctors need statistics? Doctors’ use of and attitudes to probability and statistics. Stat Med. 2009;28(15):1969–81. 10.1002/sim.3608. 19452567 10.1002/sim.3608

[11] Arias A, Peters OA, Broyles IL. New curricular design in biostatistics to prepare residents for an evidence-based practice and lifelong learning education: a pilot approach. Int Endod J. 2017;50(10):999–1010. 10.1111/iej.12714. 27783428 10.1111/iej.12714

[12] McCullough JPA, Lipman J, Presneill JJ. The statistical curriculum within randomized controlled trials in critical illness. Crit Care Med. 2018;46(12):1985–90. 10.1097/CCM.0000000000003380. 30119072 10.1097/CCM.0000000000003380

[13] Kern DE, Thomas PA, Hughes MT. Curriculum development for medical education: a six‐step approach. 2nd ed. Baltimore, Maryland: Johns Hopkins University Press; 2010.

[14] Wiggins GP, McTighe J. The understanding by design guide to creating high-quality units. Alexandria, Va: ASCD; 2011.

[15] Fischer C. Master the Boards USMLE Step 3. 5th ed. Kaplan Publishing; 2018.

[16] Mikulich SK, Zerbe GO, Jones RH, Crowley TJ. Relating the classical covariance adjustment techniques of multivariate growth curve models to modern univariate mixed effects models. Biometrics. 1999;55(3):957–64. 10.1111/j.0006-341X.1999.00957.x. 11315035 10.1111/j.0006-341x.1999.00957.x

[17] Schnurr PP. Assessing psychotherapy: practical examples. In: Boutron I, Ravaud P, Moher D, editors. Randomized clinical trials of nonpharmacological treatments. New York: CRC Press; 2012. pp. 325–337.

[18] Himelhoch S, Edwards S, Ehrenreich M, Luber MP. Teaching lifelong research skills in residency: implementation and outcome of a systematic review and meta-analysis course. J Grad Med Educ. 2015;7(3):445–50. 10.4300/JGME-D-14-00505.1. 26457153 10.4300/JGME-D-14-00505.1 PMC4597958

[19] Worley E, Suh EH, Abrukin L, DeFilippo M, Kamler JJ, Polavarapu M, et al. Harnessing residents’ practice-based inquiries to enhance research literacy: the thoughtful reading of evidence into clinical settings (T-RECS) initiative. West J Emerg Med. 2025;26(3):564–8. 10.5811/westjem.20921. 40561957 10.5811/westjem.20921 PMC12208079

[20] Roberts D. The engagement agenda, multimedia learning and the use of images in higher education lecturing: or, how to end death by PowerPoint. J Furth High Educ. 2017;42(7):969–85. 10.1080/0309877X.2017.1332356.

